# Effects of Ankle Muscle Fatigue and Visual Behavior on Postural Sway in Young Adults

**DOI:** 10.3389/fphys.2019.00643

**Published:** 2019-06-05

**Authors:** Fabio A. Barbieri, Tiago Penedo, Lucas Simieli, Ricardo A. Barbieri, Alessandro M. Zagatto, Jaap H. van Diëen, Mirjam A. G. M. Pijnappels, Sérgio T. Rodrigues, Paula F. Polastri

**Affiliations:** ^1^ Human Movement Research Laboratory (MOVI-LAB), Graduate Program in Movement Science, Department of Physical Education, São Paulo State University (UNESP), São Paulo, Brazil; ^2^ Graduate Program in Physical Education and Sport, School of Physical Education and Sport of Ribeirao Preto (EEFERP), Centro Universitário Estácio de Ribeirão Preto, University of São Paulo, São Paulo, Brazil; ^3^ Laboratory of Physiology and Human Performance (LAFIDE), Graduate Program in Movement Science, Department of Physical Education, São Paulo State University (UNESP), São Paulo, Brazil; ^4^ Department of Human Movement Sciences, Research Institute Amsterdam Movement Sciences, Vrije Universiteit Amsterdam, Amsterdam, Netherlands; ^5^ Laboratory of Information, Vision and Action (LIVIA), Graduate Program in Movement Science, Department of Physical Education, São Paulo State University (UNESP), São Paulo, Brazil

**Keywords:** fatigue, posture, vision, saccadic eye movements, human movement, sensorial integration

## Abstract

Ankle muscle fatigue has been shown to increase body sway. In addition, body sway in quiet upright standing is reduced when saccadic eye movements are performed. The purpose of this study was to investigate the effects of visual information manipulation on postural control during ankle muscle fatigue in young adults. Twenty young adults performed: (1) two 60-s trials in quiet bipedal standing with eyes open, eyes closed, and while performing saccadic eye movements; (2) maximum voluntary isometric contractions in a leg press device, custom-made to test ankle plantar flexion force; (3) a calf raise exercise on top of a step to induce ankle muscle fatigue; and (4) a repetition of items 1 and 2. Postural sway parameters were compared with two-way ANOVAs (vision condition × fatigue; *p* < 0.05). Ankle muscle fatigue increased anterior-posterior and medial-lateral displacement and RMS of sway, as well as sway area. Saccadic eye movements reduced anterior-posterior displacement and RMS of sway and area of sway compared to eyes open and eyes closed conditions. Both saccadic eye movements and eyes closed increased the frequency of AP sway compared to the eyes open condition. Finally, anterior-posterior displacement, anterior-posterior RMS, and both anterior-posterior and medial-lateral sway frequency were affected by an interaction of fatigue and vision condition. Without muscle fatigue, closing the eyes increased anterior-posterior displacement and RMS of sway, compared to eyes open, while during muscle fatigue closing the eyes closed reduced anterior-posterior displacement and had no significant effect on anterior-posterior RMS. In conclusion, body sway was increased after induction of ankle muscle fatigue. Saccadic eye movements consistently reduced postural sway in fatigued and unfatigued conditions. Surprisingly, closing the eyes increased sway in the unfatigued condition but reduced sway in the fatigued condition.

## Introduction

Postural control is essential for the performance of daily activities. Postural oscillations (postural sway) need to be controlled because they may interfere with performance and large oscillations could even contribute to the loss of balance. Recent studies showed that saccadic eye movements can positively affect postural control (Rodrigues et al., 2013, 2015; Bonnet and Baudry, 2016a). When saccadic movements are performed during standing, postural sway is reduced to allow accurate gaze shifts, which is an indication of functional integration of posture and gaze control (Stoffregen et al., 2006). This integration is attained by afferent (minimizing the changes of the projected image on the retina) and efferent (attenuating postural sway in an attempt to connect pre- and post-saccadic views of the scene) mechanisms (Guerraz and Bronstein, 2008). The reduction of postural sway has practical implications for performance in standing tasks (Zemková and Hamar, 2014).

Previous studies have conclusively shown that ankle (calf) muscle fatigue increases postural sway (Vuillerme et al., 2001, 2006; Boyas et al., 2011, 2013). The effects of ankle muscle fatigue on postural control have been explained by impairments of somatosensory input (Gribble and Hertel, 2004b), such as decreased position sense acuity (Björklund et al., 2000) and reduced neural transmission (Qu et al., 2009). As muscle fatigue directly affects somatosensory information (Gribble and Hertel, 2004a,b), this may be discarded by the postural control system at a central level during muscle fatigue, causing increased reliance on visual information (Vuillerme et al., 2001, 2006; Ledin et al., 2004). Previous studies have shown that visual information may compensate for the destabilizing effects of ankle muscle fatigue during quiet standing (Vuillerme et al., 2001, 2006; Ledin et al., 2004; Boyas et al., 2011, 2013). On the other hand, the evidence on the negative effects of the absence of vision on postural control (greater postural sway) during ankle muscle fatigue is inconclusive (Corbeil et al., 2003). Saccadic eye movement may serve as a strategy to increase postural stability under ankle muscle fatigue. Understanding whether saccadic eye movements provide a short-term solution for improving postural control under ankle muscle fatigue can contribute to developing strategies to combat balance impairments.

Assuming that (1) the ankle muscle fatigue increases postural sway due to impaired proprioception, (2) vision can reduce the increase in postural sway with ankle muscle fatigue, and (3) postural sway in quiet upright standing is reduced when saccadic eye movements are performed, the purpose of the present study was to investigate the effects of visual information manipulation (eyes closed, eyes open with fixed target, and eyes open with saccadic eye movements) on postural sway during ankle (calf) muscle fatigue in young adults. We hypothesized that the effects of saccadic eye movements and eye closed are stronger in the fatigued than in the unfatigued condition. Specifically, saccadic eye movements will alleviate the effects of ankle muscle fatigue, reducing postural sway compared to a condition with eyes open with a fixed gaze direction, while closing the eyes will increase postural sway compared to a condition with eyes open with a fixed gaze direction under ankle muscle fatigue. In addition to parameters reflecting the magnitude of sway, we analyzed the frequency content of the sway signals, as this may provide some insight into motor strategies used.

## Materials and Methods

### Participants

Twenty young male adults (1.74 ± 0.06 m: 1.60–1.86 m; 75.97 ± 12.91 kg: 58.70–108.90 kg; 24 ± 3 years old: 20–30 years old) participated in this study. Exclusion criteria were the use of drugs that interfere with postural control (e.g., cardiovascular medication, antidepressants, benzodiazepines, opioids, and diuretics), self-report of musculoskeletal and/or neuromuscular impairments in the previous 6 months, and impairments in visual acuity not corrected by lenses. This study was carried out in accordance with the recommendations of Helsinki declaration, Ethics Committee on Human Research of the São Paulo State University, Bauru. The protocol was approved by the Ethics Committee on Human Research of the São Paulo State University, Bauru (CAAE: #48439015.0.0000.5398). All subjects gave written informed consent in accordance with the Declaration of Helsinki.

### Experimental Protocol

The participants were instructed not to perform any strenuous physical activity in 48 h before the evaluation. They performed a warm-up with walking and stretching of 5 min before the start of the experimental protocol. In addition, they performed a series of familiarization trials (three or four trials) in the leg press instrument before the maximum voluntary isometric contractions (MVICs).

The following sequence of tasks was performed: (1) postural control protocol; (2) MVIC protocol; (3) ankle muscle fatigue protocol; (4) postural control protocol; and (5) MVIC protocol. No rest period was allowed between trials, and testing was started as soon as possible after the fatigue protocol, and the time between the fatigue protocol and the postural control trials (<6 min) was expected not to allow fatigue recovery (Barbieri et al., 2016).

#### Postural Control Protocol

The participants were tested in quiet bipedal (side-by-side) standing, barefoot, on a single force plate [AccuGait, Advanced Mechanical Technologies Inc. (AMTI), Boston, MA, USA], 50 cm × 50 cm, collecting data at a sample rate of 200 samples/s. They placed their feet side-by-side and shoulder-width apart, and the position of their feet was reproduced in all subsequent trials, by drawing their foot contours on a paper sheet fixed to the force plate. Two 60-s trials, before and during ankle muscle fatigue, of the following postural control conditions were performed: (1) eyes open (EO): quiet standing with gaze fixation on a stationary target positioned in front of the participant; (2) eyes closed (EC): quiet standing with closed eyes; and (3) saccadic eye movements (SE): quiet standing, performing saccades directed to a target appearing on one side of a monitor, then disappearing and reappearing simultaneously on the opposite side of the monitor once per 2 s. The monitor was positioned 1 m away from the participant’s eyes. The target was a red dot, 2 cm in diameter, on a white background with a subtended visual angle of approximately 1.15°. The total distance (19.5 cm) between right and left targets comprised a visual angle of 11° to avoid head movements (Rodrigues et al., 2015). Stimuli were generated by Flash Mx software (Macromedia) and presented on an LCD monitor (37.5 cm × 30 cm, LG, Faltron L1952H, 50/60 Hz, 0.8A). The order of the trials was randomized for each participant. For all trials, the participants were instructed to stand quietly in an upright position. In addition, in the EO and SE, the participants were instructed to keep their gaze on the target. An experienced researcher ensured that the participants remained quiet and complied with gaze instructions.

The center of pressure (CoP) was determined from the ground reaction forces by means of moment-of-force equilibrium calculations. The first 10 s of each recording were ignored to avoid potential disturbances resulting from delayed stabilization after the participant stepped onto the force plate. The data were filtered with a fourth-order low-pass Butterworth filter with a cut-off frequency of 5 Hz. Total displacement and root mean square (RMS), expressing CoP movement, were calculated for the anterior-posterior (AP) and medial-lateral (ML) directions, separately. In addition, the sway area (area of an ellipse containing 95% of the CoP data) and finally the median frequency of sway were calculated (Duarte and Freitas, 2010). The last parameter was calculated by employing spectral analysis of the position time series, separately for both movement directions.

Gaze behavior was recorded with a mobile eye tracker (Mobile Eyes-5 glasses, ASL, Bedford, MA, USA) during EO and SE. The data acquisition rate was 60 samples/s. The eye tracker system was calibrated using the nine-point calibration method. Calibration was also checked periodically between trials. Gaze fixation was defined as the stabilization of gaze (when two times points of gaze standard deviation—95% confidence interval—were less than one degree of visual horizontal and vertical angles) over 99 ms (Rodrigues et al., 2016; Gotardi et al., 2019; Santinelli et al., 2019). We analyzed the following parameters: number of fixations, mean duration of fixations, normalized total duration of fixations (the sum of fixations divided by the duration of the trial), and the area of fixation displacement (area of an ellipse that contained 85% of the horizontal and vertical fixation position data; Rodrigues et al., 2016). The area of fixation displacement for SE was analyzed separately for the right and left targets.

#### Maximum Voluntary Isometric Contraction Protocol

The MVIC was performed in a leg press device, custom-made to test ankle plantar flexion. A load cell with a precision of 0.1 kgf was used in combination with a signal amplifier (CSA/ZL-100Kgf, MK Control, São Paulo, Brazil) to collect force data. The force data were acquired using Labview software (National Instruments Inc., Austin, TX, USA) at a rate of 1,000 samples/s. The participants were seated in a backward inclined chair, with the hip joints flexed 90°, knee joints fully extended, and the feet in a neutral position with only the distal half of the feet contacting the load cell. The position of the feet on the device was marked to maintain the same position over the trials. The joint angles were determined by a mechanical goniometer. The participants were firmly secured with straps fastening legs and shoulders. The participants performed the task with both legs, with the instruction to produce maximum force as fast as possible without flexing the knee joint. Total contraction duration was 5 s. Participants performed two attempts before and after the ankle muscle fatigue protocol, with 2 min rest between attempts. The participants were verbally encouraged to perform the muscle contractions. The MVIC was determined as the mean of the peak values in the two attempts before and after the muscle fatigue protocol.

#### Ankle Muscle Fatigue Protocol

**Figure 1:**
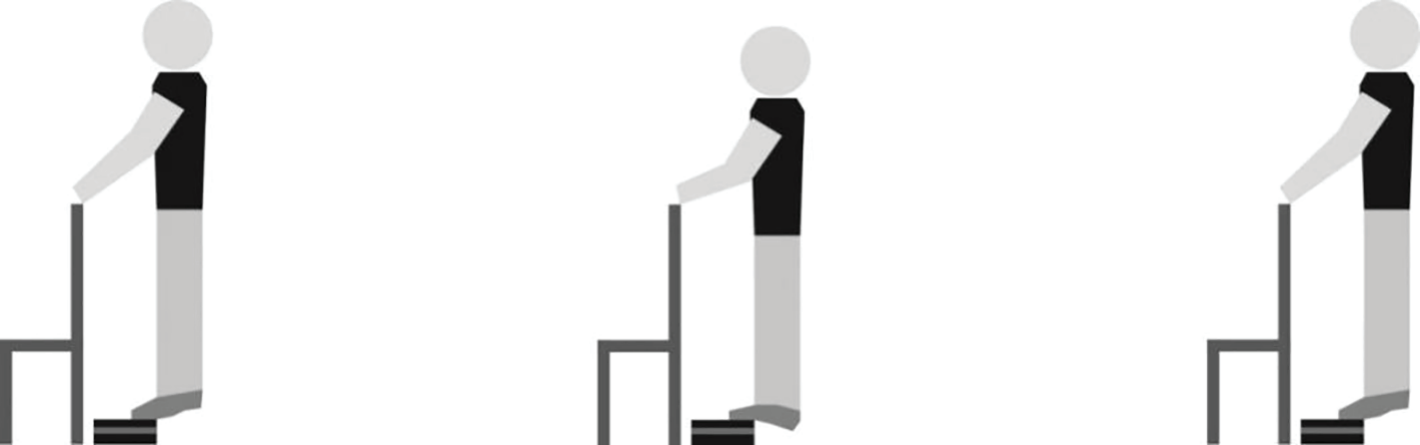
Schematic representation of the ankle muscle fatigue protocol.

Muscle fatigue was induced by a calf raise exercise performed standing on top of a step (Figure 1; Barbieri et al., 2013). The speed of the exercise was controlled by a metronome at 30 beats/min. Initially, the participants performed some practice trials. The instruction was to repeatedly perform plantar flexion and dorsiflexion of the ankle over the complete range of motion at the frequency of movement indicated by the metronome. Participants were allowed to touch the back of a chair with their hands to ensure balance. The fatigue protocol was stopped when participants indicated to be unable to continue, or when they reduced the range of ankle plantar flexion compared to the beginning of the protocol, or when they no longer performed at the desired movement frequency after encouragement. To rate the level of fatigue, a 20-point Borg scale (Borg, 1982) was filled in by the participants before and immediately after the muscle fatigue protocol. The endurance time during the fatigue protocol was recorded.

### Statistical Analysis

The dependent variables were statistically analyzed with SPSS 15.0 for Windows (*α* < 0.05). The data were normally distributed and verified by the Shapiro--Wilk test. The body sway parameters were compared through two-way ANOVAs (fatigue: before and after the muscle fatigue protocol; vision: EO, EC, and SE) with repeated measures for both factors. Tukey’s *post hoc* tests were used to find differences among levels. The MVIC parameter and gaze parameters (separately for EO and SE) were analyzed with a Student’s *t* test for repeated measurements, to assess the effect of fatigue. Partial *η*^2^ was reported to express effect size and interpreted as small (*η*^2^ > 0.01), medium (*η*^2^ > 0.06), or large (*η*^2^ > 0.14; Cohen, 1988).

## Results

### Maximum Voluntary Isometric Contraction and Fatigue Protocol

The ankle muscle fatigue protocol lasted on average 270 s (±188 s – range: 94–955 s). The individuals reported a Borg score of 18 (±2–range: 15–20) after the fatigue protocol. In addition, the MVIC was reduced by 7% (range: 3–15%; *t*_19_ = 2.25, *p* < 0.032; before the fatigue protocol: 809 ± 231 N; after the fatigue protocol: 754 ± 188 N).

### Postural Sway

The mean values, standard deviations, *p*’*s*, and effect sizes of CoP parameters in EO, EC, and SE before and after the muscle fatigue protocol are presented in Table 1. Main effects of fatigue on all parameters expressing amplitude of sway were found. In addition, vision conditions (main effects of vision condition) affected all AP amplitude parameters and frequency of sway in both directions. Finally, AP displacement, AP RMS, and both AP and ML sway frequency were affected by an interaction of fatigue and vision condition. In the next paragraph, we first present the significant fatigue and vision condition interaction effects and subsequently the significant main effects of fatigue and vision condition.

**Table 1:** Mean values and standard deviations of the center of pressure parameters and gaze parameters according to conditions before and after the fatigue protocol.

		Unfatigued condition			Fatigued condition			*p*		
		Eyes open	Saccadic eye movements	Eyes closed	Eyes open	Saccadic eye movements	Eyes closed	Fatigue	Condition	Interaction
Body sway parameters	AP displacement (cm)	305.32 ± 100.38	233.09 ± 82.65	347.89 ± 112.36	448.49 ± 169.80	311.89 ± 133.01	378.30 ± 100.44	**0.001** (0.46)	**0.001** (0.44)	**0.036** (0.17)
	ML displacement (cm)	111.89 ± 24.12	111.42 ± 20.79	111.95 ± 17.63	131.85 ± 22.39	125.92 ± 32.10	127.25 ± 27.84	**0.002** (0.41)	0.750	0.687
	AP RMS	0.38 ± 0.11	0.28 ± 0.09	0.43 ± 0.13	0.56 ± 0.21	0.39 ± 0.15	0.49 ± 0.13	**0.001** (0.48)	**0.001** (0.45)	**0.047** (0.15)
	ML RMS	0.14 ± 0.03	0.15 ± 0.04	0.14 ± 0.02	0.16 ± 0.02	0.16 ± 0.04	0.17 ± 0.04	**0.006** (0.33)	0.777	0.606
	Area (cm^2^)	1.04 ± 0.42	0.78 ± 0.33	1.09 ± 0.35	1.72 ± 0.84	1.27 ± 0.68	1.57 ± 0.60	**0.001** (0.65)	**0.005** (0.27)	0.509
	AP median frequency (Hz)	0.69 ± 0.18	1.27 ± 0.62	0.96 ± 0.43	0.67 ± 0.25	1.02 ± 0.55	1.11 ± 0.54	0.490	**0.001** (0.36)	**0.038** (0.15)
	ML median frequency (Hz)	2.19 ± 0.92	2.14 ± 1.04	2.40 ± 0.84	1.53 ± 0.47	2.25 ± 1.59	2.44 ± 1.19	0.278	**0.042** (0.16)	**0.043** (0.14)

P-values refers to main effects of fatigue and condition and interaction effects between fatigue and condition. Significant effect sizes are given in parentheses. AP, anterior-posterior; ML, medial-lateral; RMS, root mean square.

**Figure 2:**
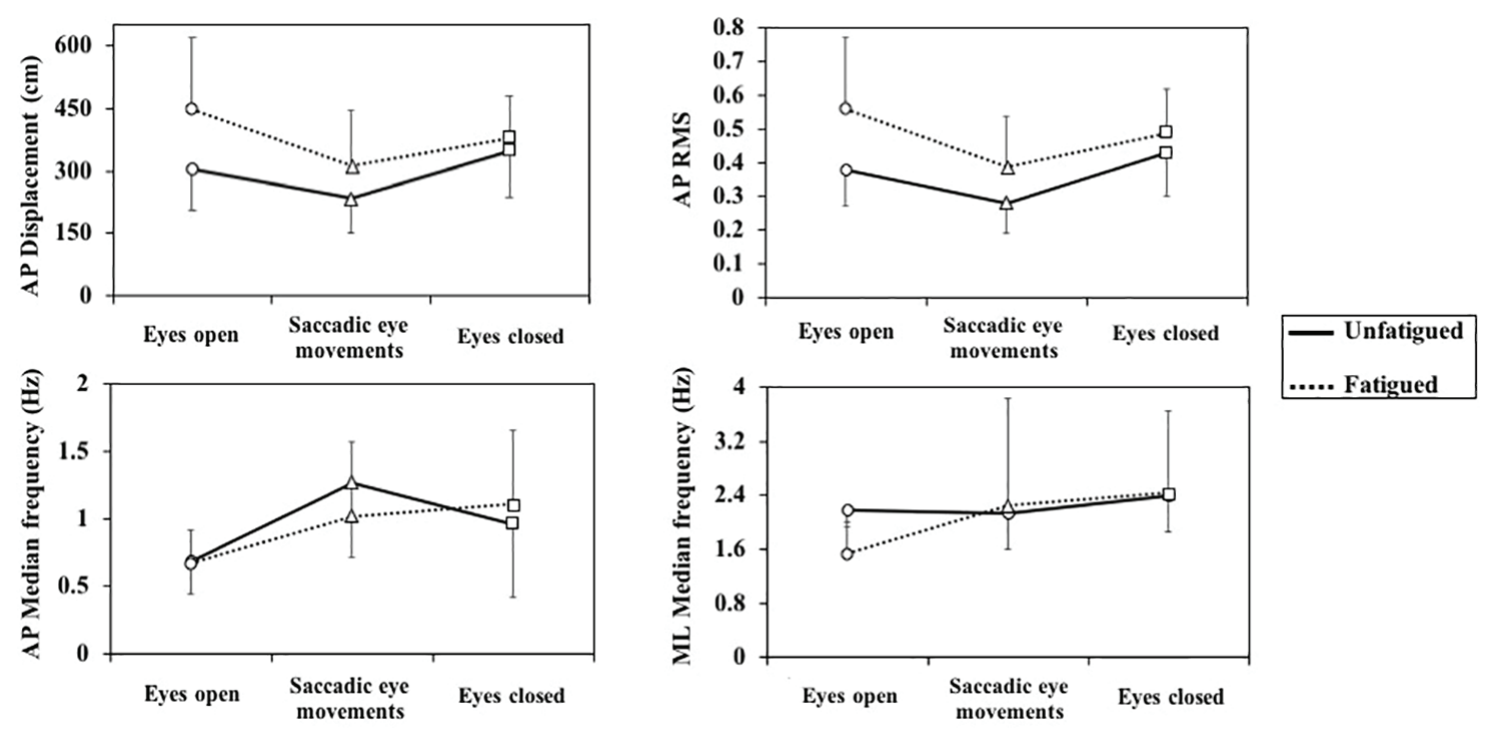
Fatigue × vision interactions for center of pressure parameters. AP, anterior-posterior; ML, medial-lateral.

ANOVAs indicated interaction effects between fatigue and vision condition (Figure 2) on AP displacement (*F*_2,32_ = 3.93) and RMS (*F*_2,32_ = 3.33) of sway. *Post hoc* tests indicated that SE reduced AP displacement and RMS in the fatigued condition (*p* = 0.005 and *p* = 0.003, respectively) and in the unfatigued condition (*p* = 0.006 and *p* = 0.006, respectively), compared to the EO fixed gaze condition. Without muscle fatigue, closing the eyes increased AP displacement and RMS of sway (*p* = 0.041 and *p* = 0.050, respectively), compared to EO, while during muscle fatigue closing the eyes reduced AP displacement (*p* = 0.048) and had no significant effect on AP RMS (*p* = 0.096). Ankle muscle fatigue had no effects on AP displacement and RMS in the EC (*p* = 0.133 and *p* = 0.056, respectively), and it increased AP displacement and RMS with EO (*p* = 0.003 and *p* = 0.002, respectively) and with SE (*p* = 0.011 and *p* = 0.026, respectively). Overall, these results suggest less visual dependence in the fatigued state and higher sensitivity to fatigue with eyes open, in contrast to our hypothesis.

With respect to interaction effects between fatigue and vision condition on the sway frequency, *post hoc* tests indicated that SE coincided with increased frequency of AP sway in the unfatigued and in the fatigued conditions (*p* = 0.001 and *p* = 0.016) compared to the EO fixed gaze condition. Closing the eyes similarly increased AP sway frequency in the unfatigued and fatigued conditions (*p* = 0.018 and *p* = 0.002) compared to the EO. The frequency of ML sway increased during SE (*p* = 0.035) and closing the eyes (*p* < 0.001) relative to the EO fixed gaze condition in the fatigued condition only. A significant *post hoc* effect of fatigue on frequency content was found only for the ML direction in the EO, where fatigue coincided with increased frequency (*p* < 0.001).

**Figure 3:**
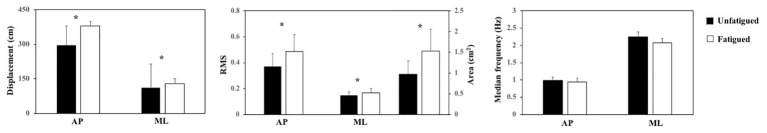
Means and standard deviations (main effect of fatigue) of the center of pressure parameters before and after ankle muscle fatigue. AP, anterior-posterior; ML, medial-lateral. *Indicates a difference between before and after ankle muscle fatigue.

The main effect of fatigue is presented in Figure 3. Ankle muscle fatigue increased AP and ML displacement (*F*_1,19_ = 16.44 and *F*_1,19_ = 12.99, respectively) and RMS of sway (*F*_1,19_ = 17.99 and *F*_1,19_ = 9.40, respectively), as well as sway area (*F*_1,19_ = 36.59).

**Figure 4:**
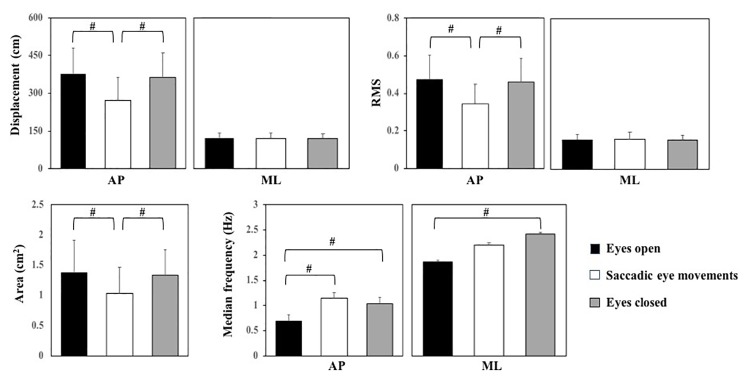
Means and standard deviations (main effect of vision conditions) of the center of pressure parameters for eyes open, saccadic eye movements, and eyes closed condition. AP, anterior-posterior; ML, medial-lateral. #Indicates a significant *post hoc* difference between conditions.

The main effect of vision conditions is presented in Figure 4. The vision conditions affected AP displacement (*F*_2,34_ = 15.04) and RMS (*F*_2,34_ = 15.73) of sway and area of sway (*F*_2,32_ = 7.03). In line with our hypothesis, SE reduced AP displacement and RMS of sway and area of sway compared to EO (*p* < 0.001) and EC (*p* < 0.01). However, in contrast to our hypothesis, differences between EO and EC were not significant. Vision condition also affected AP (*F*_2,38_ = 10.83) and ML sway frequency (*F*_2,33_ = 3.67). Both saccadic eye movements and closing the eyes increased the frequency of AP sway compared to the EO (*p* < 0.001 and *p* < 0.005, respectively). ML sway frequency was higher with eyes closed than with eyes open (*p* = 0.006).

### Effects of Muscle Fatigue on Gaze Behavior

In the fatigued EO condition, the number of fixations was higher (*t*_19_ = −2.32), and the mean duration of fixations was lower (*t*_19_ = 2.67) compared to the unfatigued EO condition (Table 2). In addition, the variability of the location of fixations was larger in the fatigued condition (*t*_19_ = −2.68). There were no effects of muscle fatigue on eye movement parameters in the SE (*p* > 0.05).

**Table 2:** Means and standard deviations of gaze parameters for eyes open and saccadic eye movements before and after ankle muscle fatigue.

		Unfatigued condition			Fatigued condition		
		Eyes open	Saccadic eye movements	Eyes closed	Eyes open	Saccadic eye movements	Eyes closed
Gaze parameters	Number of fixation (*n*)	29.91 ± 19.14 **(*p* < 0.03, 0.15)**	111.57 ± 24.40 (*p* = 0.64)	–	35.78 ± 16.84	113.02 ± 16.49	–
	Mean duration of the fixations (s)	3.12 ± 1.89 **(*p* < 0.01, 0.24)**	0.47 ± 0.10 (*p* = 0.74)	–	2.12 ± 1.35	0.47 ± 0.07	–
	Normalized total duration of fixations (%)	95.68 ± 4.14 (*p* = 0.21)	84.94 ± 10.33 (*p* = 0.31)	–	93.36 ± 7.41	87.26 ± 6.09	–
	Area of fixation displacement (pixels)	579.08 ± 350.30 **(*p* = 0.015, 0.27)**	RS: 1238.01 ± 674.86 (*p* = 0.88) LS: 1199.74 ± 546.47 (*p* = 0.49)	–	934.08 ± 697.92	RS: 1220.96 ± 688.64 LS: 1302.17 ± 773.36	–

RS, right side; LS, left side. The P-values and significant effect sizes are given in parentheses.

## Discussion

We investigated the effects of visual information manipulations and ankle muscle fatigue on postural sway in young adults. The calf muscles are considered to play a major role in postural control (Panzer et al., 1995), and in line with this hypothesis, ankle muscle fatigue increased postural sway, in agreement with previous studies (Vuillerme et al., 2001, 2006; Boyas et al., 2011, 2013). We showed reduced ankle muscle strength after the fatigue protocol, which may have impaired neuromuscular control of upright posture (Gribble and Hertel, 2004a), as it may have limited the ability to produce or sustain the required force output of the ankle muscles for stabilization of the upright posture (Ledin et al., 2004; Vuillerme et al., 2006). In addition to impairments in muscle contractile efficiency, decreased proprioceptive acuity may have contributed to impaired control (Gribble and Hertel, 2004a; Boyas et al., 2011). Ankle muscle fatigue affected the gaze behavior in the EO condition. A higher number of fixations, reduced mean duration of fixations, and more variability in location of the fixations may suggest reduced attention to the visual task in the fatigued condition (Stoffregen et al., 2006; Bonnet and Baudry, 2016b) but may also reflect that increased sway interferes with gaze fixation on a stationary target. These effects of muscle fatigue on postural and gaze behavior can be explained by physiological mechanisms, which are characterized by peripheral disturbances at the level of the active muscles, and the central nervous system fails to drive the motoneurons adequately (Gandevia, 2001).

The novelty of this study was that the interaction effects of vision and fatigue were not consistent with our hypothesis. Saccadic eye movements did reduce sway similarly in the fatigued and unfatigued conditions, and closing the eyes only affected sway in the unfatigued condition. So, in contrast to our hypothesis, these results indicate overall reduced rather than increased effects of visual information with fatigue. The attenuation of postural sway during SE in ankle muscle fatigue suggests that fatigue did not reduce the benefit of an increased retinal flow caused by saccades for postural control (Rodrigues et al., 2015). Although we did not measure any physiological parameter, the literature is consistent to indicate that muscle fatigue causes central and peripheral disturbances, such as decreased somatosensory input (Gribble and Hertel, 2004b) and reduced neural transmission (Qu et al., 2009). However, our results suggest that physiological disturbances may not enough to impair the functional integration between posture and gaze control, which partially compensate the effects of ankle muscle fatigue on body sway. Therefore, using eye saccadic movement may serve as a strategy for increasing postural stability under ankle muscle fatigue. In addition, SE coincided with reduced postural sway in comparison to the EO and EC conditions. Similarly, previous studies indicated that although saccades briefly suppress visual perception, they coincide with improved postural control (Stoffregen et al., 2006; Rodrigues et al., 2013, 2015). Possibly, SE requires greater postural stability to allow more accurate gaze shifts (Stoffregen et al., 2006). Alternatively, postural control during saccadic eye movements may improve, as it becomes more automatic and regulated by lower-level structures, such as the cerebellum or basal ganglia (Bonnet and Baudry, 2016a). We observed an increase in the frequency of AP sway when performing saccadic eye movements, which may reflect a compensatory strategy to deal with the reduced sensory input during the saccades, as discussed above. In addition, the saccadic movement reduced visual information, which can lead to stiffening of the ankle joints through cocontraction, causing reduced and higher frequency of sway.

Surprisingly and in contrast to our hypothesis, closing the eyes increased body sway relative to the eyes open with fixed gaze direction condition only in the unfatigued condition. The absence of this effect in the fatigued condition contrasts with quite consistent effects of closing the eyes on postural sway reported in the literature (Winter, 1995; Agostini et al., 2016). However, vision has been estimated to account only for 10% of the sensory input used for balance control when standing (Peterka, 2002). Fatigue is thought to affect sensory information mainly through a change in muscle spindle thresholds (Corbeil et al., 2003). This may suggest that information from other modalities (e.g., foot sole pressure and vestibular information) could compensate for these effects, without increasing visual dependence. In turn, this would suggest that ankle muscle fatigue affects balance more through its effects on motor output of the postural control system than through its effects on the sensory estimates of body orientation. In the present experiment, subjects were found to increase the frequency of postural sway when closing their eyes, which may reflect lower thresholds of postural responses in intermittent feedback control (Tanabe et al., 2016), increased gains in continuous feedback control (Maurer and Peterka, 2005; Pruszynski and Scott, 2012), or increased stiffness through increased tonic ankle muscle contraction (Ledin et al., 2004; Bonnet, 2016). The latter could be a compensatory strategy to deal with the reduced sensory input when closing the eyes, especially in the presence of fatigue. In this context, it is important to note that sway is not minimized in a normal upright stance and be reduced at the cost of somewhat increased muscle activity (Houdijk et al., 2015). Since tonic muscle activity in bipedal stance is low, this strategy might still be feasible in the fatigued condition. However, we did not observe a consistent increase in sway frequency with fatigue.

The muscle fatigue protocol used in the present study allowed for variability in exertion, and endurance was quite variable between participants. However, all participants reported a hard to extremely hard level of exertion during the fatigue protocol, and MVIC was reduced after the fatigue protocol in all of them. This would suggest that levels of fatigue were comparable. The findings cannot be generalized to different fatigue levels or to fatigue in other muscle groups involved in postural control.

In conclusion, postural sway increased after induction of ankle muscle fatigue. Saccadic eye movements consistently reduced postural sway in fatigued and unfatigued conditions. Surprisingly, closing the eyes increased sway in the unfatigued condition but reduced sway in the fatigued condition. The implication of these findings that individuals can adjust sensory weights to improve postural control after ankle muscle fatigue, but these adjustments are dependent upon vision conditions. This suggests that when tasks, which require stability and accuracy, are performed under muscle fatigue, the quality of visual information can positively influence postural control and, consequently, task performance. Eye movement or vision manipulations may serve as a strategy for increasing postural stability under ankle muscle fatigue.

## Ethics Statement

The protocol applied in this study was approved by the local Research Ethics Committee (#48439015.0.0000.5398).

## Author Contributions

FB, TP, and LS designed the study. All authors edited the manuscript. TP, LS, and RB did the experiment and analyzed the data. AZ, SR, PP, JD, FB, and MP contributed to the interpretation of the results and drafted the manuscript. FB, TP, and JD performed the statistical analysis. FB, SR, and PP administrated this project.

### Conflict of Interest Statement

The authors declare that the research was conducted in the absence of any commercial or financial relationships that could be construed as a potential conflict of interest.
